# The Intersection of the Oral Microbiome and Salivary Metabolites in Head and Neck Cancer: From Diagnosis to Treatment

**DOI:** 10.3390/cancers16203545

**Published:** 2024-10-21

**Authors:** Maria Gonzalez Agurto, Nicolas Olivares, Gisela Canedo-Marroquin, Daniela Espinoza, Sofia C. Tortora

**Affiliations:** 1 Faculty of Dentistry, Universidad de los Andes, Santiago 7620086, Chile; gcanedo@uandes.cl; 2 Faculty of Dentistry, Pontificia Universidad Católica de Chile, Santiago 8330024, Chile; nholivares@uc.cl; 3 Millennium Institute on Immunology and Immunotherapy (MIII), Santiago 8331150, Chile; 4 Faculty of Dentistry, Universidad Mayor, Santiago 8580745, Chile; 5 Immunology Program, Memorial Sloan Kettering Cancer Center, New York, NY 10065, USA

**Keywords:** head and neck cancer, oral microbiota, saliva, radiotherapy, metabolites, mucin, dysbiosis

## Abstract

Head and neck cancers (HNCs) are the seventh most common cancer worldwide, accounting for 4–5% of all malignancies. Salivary metabolites, which serve as key metabolic intermediates and cell-signalling molecules, are emerging as potential diagnostic biomarkers for HNC. While current research has largely concentrated on these metabolites as biomarkers, a critical gap remains in understanding their fluctuations before and after treatment, as well as their involvement in oral side effects. Recent studies emphasise the role of the oral microbiome and its metabolic activity in cancer progression and treatment efficacy by bacterial metabolites and virulence factors. Oral bacteria, such as *P. gingivalis* and *F. nucleatum*, contribute to a pro-inflammatory environment that promotes tumour growth. Additionally, *F. nucleatum* enhances its virulence through flagellar assembly and iron transport mechanisms, facilitating tumour invasion and survival. Moreover, alterations in the oral microbiome can influence chemotherapy efficacy and toxicity through the microbiota–host irinotecan axis, highlighting the complex interplay between microbial communities and therapeutic outcomes. Salivary metabolite profiles are influenced by factors such as gender, methods, and patient habits like smoking—a major risk factor for HNC. Radiotherapy (RT), a key treatment for HNC, often causes side effects such as xerostomia, oral mucositis, and swallowing difficulties which impact survivors’ quality of life. Intensity-modulated radiotherapy (IMRT) aims to improve treatment outcomes and minimise side effects but can still lead to significant salivary gland dysfunction and associated complications. This review underscores the microbial and host interactions affecting salivary metabolites and their implications for cancer treatment and patient outcomes.

## Simple Summary

Head and neck cancers (HNCs) represent 4–5% of all malignancies globally. Salivary metabolites, as metabolic intermediates and signalling molecules, are gaining attention as diagnostic biomarkers for several diseases, including HNC; however, the metabolites’ role in cancer treatment outcomes and oral side effects remains underexplored. Current studies focus on the oral microbiome’s significance in promoting a pro-inflammatory environment that facilitates tumour development and invasion. Specific microorganisms and their metabolites influence chemotherapy efficacy through several mechanisms. Radiotherapy (RT), a standard HNC treatment, can modify the oral microbiota and salivary metabolite profiles, leading to a wide range of side effects. To reduce oral complications, intensity-modulated radiotherapy (IMRT) was developed; however, cancer survivors often experience a reduced quality of life. This review highlights the microbial and host interactions affecting salivary metabolites and their implications for cancer treatment and patient outcomes.

## 1. Introduction 

The mouth is the first part of the digestive system and comprises structures including the teeth, tongue, and salivary glands, necessary for mastication and speech. Salivary glands produce saliva that is rich in a variety of enzymes and proteins. The digestive process commences immediately within the oral cavity, where mechanical and chemical digestion processes are initiated. Mechanical digestion involves the breakdown of food into smaller particles through mastication, which is accomplished by the action of the teeth to reduce the size of food particles. Chemical digestion, limited in the mouth compared to other sites of the gastrointestinal tract, plays a role through the enzymatic actions of saliva. Salivary amylase catalyses the hydrolysis of starches into maltose and maltotriose [1]. This enzyme operates most effectively at a pH range of 6.7 to 7.0. Lingual lipase initiates the breakdown of triglycerides by hydrolysing ester bonds to produce diacylglycerols and monoacylglycerols. Collectively, these enzymatic activities contribute to the initial stages of digestion, preparing the food for further enzymatic and chemical processing in the stomach and intestines [2].

Additionally, the oral cavity constitutes the first line of defence against pathogens. Lysozyme and lactoferrin, present in saliva, play crucial roles in antimicrobial defence. Lysozymes neutralise bacteria by breaking down the cell walls, while lactoferrin binds to iron, an essential nutrient for bacterial growth, thus inhibiting bacterial proliferation. Together, these components help maintain oral health by preventing infections and controlling the microbial environment in the mouth [3]. 

The oral epithelium comprises multiple layers of cells that function as a complex barrier with varied antigenic responses and distinct patterns of cytokeratin expression. This stratified epithelium plays a crucial role in the oral immune system, which exhibits a unique property known as oral tolerance [4]. This persistent oral tolerance is vital for preventing unnecessary immune responses to harmless dietary proteins, maintaining immune homeostasis, and preventing chronic inflammation [3].

An essential component of the oral cavity is the oral microbiome. This diverse microbial community is crucial in maintaining homeostasis by balancing microbial populations, supporting immune function, preserving barrier integrity, and influencing overall systemic health. Disruptions in this balance can lead to various oral and systemic health issues [5]. Due to its significant role, the oral microbiome of several oral cavity sites such as the saliva, tooth surfaces, mucosa, and gingival sulcus (which includes gingival and periodontal pockets) has been extensively studied using both traditional cultivation methods and advanced culture-independent molecular techniques. These include 16S rRNA gene sequencing and other methods that provide detailed insights into oral microbial diversity and function from different niches extensively. Analysis has revealed that the six most predominant phyla within the oral microbiome are *Firmicutes*, *Bacteroidetes*, *Proteobacteria*, *Actinobacteria*, *Spirochaetes*, and *Fusobacteria*. Collectively, these phyla account for approximately 96% of the total microbial taxa present in the oral cavity [6,7,8]. These findings underscore the significant diversity and complexity of the oral microbiome, highlighting the major bacterial groups that play a central role in maintaining oral health and influencing disease processes [6].

Head and neck cancers (HNCs) are the seventh most common cancer in the world, representing 4–5% of all malignancies [9]. Established lifestyle risk factors for oral cancer encompass tobacco use (both smoking and chewing), the consumption of areca nuts, excessive alcohol intake [9], and a diet deficient in antioxidant vitamins and minerals [10]. Poor oral hygiene and periodontitis, an inflammatory condition that affects the gums, have recently been identified as significant risk factors [10]. Lifestyle factors and changes in oral health can disrupt the microbial communities in specific areas of the oral cavity, altering the balance between microbes and the host and potentially contributing to the development of oral cancer [11].

Several studies have examined the association between the oral microbiome and HNC, primarily through bacterial profiling techniques. Among these, 16S rRNA gene sequencing is the most used method for analysing microbial communities [12,13,14]. Research has identified specific periodontal pathogens, such as *Porphyromonas gingivalis*, *Fusobacterium nucleate* [13,14], *Streptococcus*, *Prevotella*, and *Treponema* [15], as significant contributors to tumourigenesis. Additionally, *Fusobacterium, Treponema*, *and Leptotrichia* were enriched in oral cancer compared to non-oral cancer, such as the base of the tongue, hypopharyngeal, larynx, oropharynx, and tonsil [16]. These bacteria can stimulate cancer development by inducing the expression of molecules involved in the cancer process [13,14].

For instance, in studies using a mouse model of periodontitis induced by *P. gingivalis*, treatment led to a notable increase in free fatty acids. This change in fatty acid metabolism was associated with an increased risk of developing oral cancer, highlighting the role of *P. gingivalis* in promoting cancer through metabolic alterations [12]. Microbial dysbiosis and its metabolites in the oral cavity can be considered significant risk factors, as alterations in the microbiota contribute to key aspects of cancer development. Furthermore, specific taxa and microbial metabolites may serve as prognostic indicators when combined with pathological studies.

Radiotherapy (RT) is a core local disease control treatment for HNC patients [9,17]. Despite its effectiveness, RT is associated with numerous side effects, including salivary gland hypofunction, which impacts saliva flow and composition. Intensity-modulated radiotherapy (IMRT) was developed to enhance cancer treatment outcomes and reduce treatment-related toxicity by precisely tailoring the radiation dose to the tumour’s shape and intensity [18,19]. However, research has shown that IMRT can also lead to salivary gland dysfunction, resulting in severe hyposalivation, xerostomia, oral mucositis, and swallowing difficulties. These effects can impair daily functions, social interactions, and overall quality of life while also adding psychological and emotional burdens for cancer survivors [19]. Furthermore, reduced protective saliva functions due to these complications are linked to a decline in oral health and an increased risk of oral diseases.

Additionally, the severe lack of saliva or xerostomia can affect the integrity of both soft and hard tissues [20]. A decrease in salivary flow and changes in its composition can disrupt the spatial organisation of oral bacteria. This disruption affects several vital processes: bacterial retention, adhesion to oral surfaces, biofilm formation, and overall microbial colonisation [21]. Maintaining a stable bacterial equilibrium is vital for preventing oral infections, protecting tissue integrity, and supporting overall oral health. Thus, addressing xerostomia is essential for preserving the dynamic balance of the oral microbiome and safeguarding oral tissue health.

Furthermore, the composition of salivary proteins plays a critical role in shaping the growth and diversity of microbial communities in the oral cavity. Proteins with glycosidases, as well as those that digest glycoproteins like mucins, influence which bacteria thrive. These enzymes break down complex molecules, such as sugars found in glycoproteins, into simpler forms that can be further metabolised by other bacterial species. This biochemical conversion facilitates bacterial cooperation and helps maintain oral health by neutralising acids and regulating salivary pH [21]. Metabolites are crucial yet often less studied compared to the extensively researched salivary proteins [22]. Understanding these metabolites and their roles could provide deeper insights into microbial dynamics and oral health maintenance.

However, there is a long history of studying individual metabolites within saliva, including urea, citrate, and lactate. These metabolites have been mainly used as biomarkers for the onset and monitoring of several diseases, including cancer and renal disease. Regarding oral diseases, urea contributes to ammonia to maintain salivary pH through buffering capacity, contributing to salivary protection [23]. Despite this, the biological significance of salivary metabolites has yet to be examined in the same way as salivary proteins. Metabolites in saliva are essential molecules that originate from different sources and have numerous functions, including as metabolic intermediates of enzymatic reactions, in cell energy supply, in macromolecule synthesis, and as cell-signalling molecules that regulate metabolic pathways and other reactions and processes [24]. Much of the literature is focused on the potential biomarker role of metabolites in HNC and other pathologies [25]. However, there are fewer studies investigating the variations in these molecules and their potential role in the onset and severity of oral side effects following cancer treatment [22,26].

Several factors can influence the metabolite profile in saliva, including oral microbial imbalance, which can indicate oral inflammation or diseases [27]. Consequently, changes in the microbiome and salivary patterns often precede the onset of malignancies, highlighting the complexity of carcinogenesis, which involves multiple contributing factors. Notably, the types of metabolites found in saliva vary depending on gender, collection method, and smoking, which is a significant risk factor for HNC cancer [23]. Conversely, the salivary metabolite profile is not significantly affected by alcohol consumption, oral hygiene, medication, or food supplements. Nevertheless, there needs to be more research on the influence of the host microbiome on salivary metabolites despite the scientific community recognising the necessity for such studies [23,27].

In contrast, metabolomic studies in the gut have highlighted the crucial role of specific molecules in maintaining mucosal integrity, supporting epithelial tissue function, and regulating immune responses within the colon [28]. However, similar research remains limited in the oral environment, where microbial metabolites are known to influence the homeostasis of the oral microbiome. While bacteria significantly contribute to metabolic processes within the oral cavity, their precise role in disease pathogenesis is still not fully understood [17,23].

During the earliest studies of saliva composition in healthy individuals, it was discovered that bacteria in the oral cavity produce specific metabolites in saliva [27]. This becomes relevant in HNC patients, especially during and after cancer treatment, regarding the metabolites’ contribution to the onset and development of oral treatment side effects that impair these patients’ survival rates and quality of life [29,30]. This comprehensive review aims to explore the variation in salivary metabolites between healthy individuals and patients with head and neck conditions, both during the disease and throughout treatment.

## 2. Factors Influencing Salivary Metabolite Profile in Health and Disease

The mucosal pellicle (MP) is a thin layer, approximately 70–100 micrometres in thickness, composed of salivary proteins (mobile part) and membrane-associated mucins (MAMs) anchored to the epithelial cells [31]. It is mainly composed of two salivary mucins, which are high-molecular-weight glycoproteins, mucin 5B (MUC5B, 2–4 × 10^4^ kDa) and mucin 7 (MUC7, 130–180 kDa), secreted mainly by submandibular and minor salivary glands. Both mucins are vital in saliva viscoelastic properties regulating the lubrication, moisture, protection, and hydration of the oral mucosa. Mucins create a protective layer [32] by capturing microbial pathogens, playing an essential role in host defence.

Mucins contain glycans such as sialic acid, which microorganisms can recognise and bind, leading to spreading and selective elimination. In addition, mucins can aggregate bacteria in saliva, contributing to their clearance through swallowing. They also form a layer surrounding bacteria, maintaining their planktonic state and avoiding surface attachment [33].

**Figure 1:**
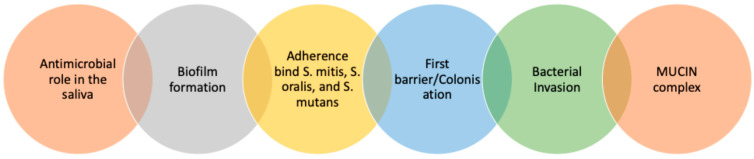
Salivary IgA’s role in saliva and pellicles contributes to selective biofilm formation by binding specific bacteria, contributing to their elimination, and maintaining oral health by preventing microbial adherence and invasion. Mucins bind IgA and transport this antibody to form an immune reservoir in the oral mucosa.

Moreover, mucins can bind to IgA, the primary oral antibody secreted by plasma cells inside the glands. Mucins transport IgA through the polymeric immunoglobulin receptor (pIgR) and serve as carriers of IgA throughout the oral cavity, physically holding and binding this protein to improve its retention in the pellicle [34]. By increasing IgA levels, the pellicle forms an immune reservoir (Figure 1) [28] to impair microbial adhesion and penetration to the epithelial cells (immune exclusion), enabling their elimination, contributing actively to the defence mechanism of the oral cavity [35,36]. IgA can aggregate bacteria to prevent mucosal colonisation and recruit others to the mucosal layer, producing changes in the oral microbiome. In this regard, bacteria involved in periodontal diseases, including *S. oralis*, *S. mutans*, *S. sanguinis*, and *S.mitior*, can cleave SIgA1 in its extended (13-amino-acid) hinge region. In addition, IgA can bind viruses, and recently, its role against SARS-CoV2 has been studied [37].

Moreover, IgA helps the bacterial colonisation of mucosal surfaces by binding certain microorganisms; however, in the gut, IgA prevents microbes from reaching the epithelium [38]. The IgA concentration increases in saliva under stress, infection, or inflammatory conditions; in contrast, there is minimal diffusion in a healthy oral environment [28,34,39,40,41]. It has been established that a reduced IgA concentration at the mucosal surfaces would impair host–microbial homeostasis, adherence, and protection from bacterial infection and toxins, thereby altering bacterial diversity and biofilm formation [42,43] and increasing the risk of infection [44,45]. This would jeopardise maintaining a healthy and functional mucosal barrier, which is critical to avoid secondary infection during epithelial disruption [46] (Figure 1).

**Figure 2:**
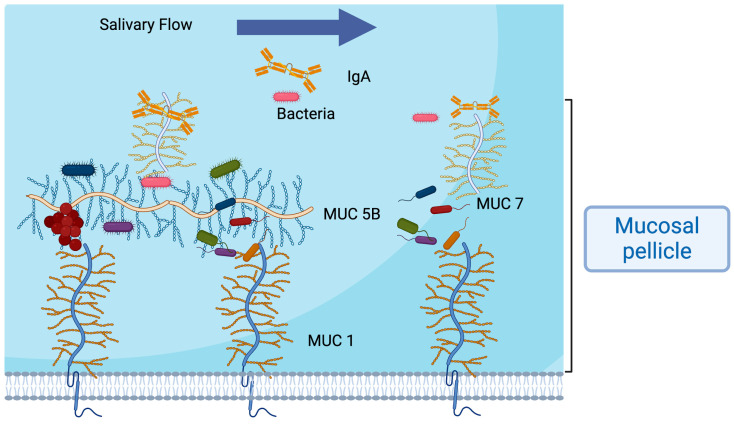
Mucosal pellicle representation, including salivary mucins, the IgA-forming complex, and mucin 1 (MUC1). Bacteria are represented by bars and discs. Mucosal pellicles contribute to bacterial adherence to epithelial cells, forming diverse colonies, maintaining homeostasis, and forming a barrier. Salivary flow rate and composition contribute to bacterial clearance by decreasing bacterial load through swallowing mechanism.

An intact mucosal pellicle plays a crucial role in maintaining oral homeostasis by preserving the integrity of oral surfaces (Figure 2). It helps reduce abrasion and chemical damage, provides immune protection, supports microbial diversity, exerts antimicrobial effects, aids in bacterial clearance, and regulates the diffusion of molecules to epithelial cells. The composition and structure of the mucosal pellicle can vary based on its location and changes in saliva flow and composition [47,48]. Additionally, alterations in the mucosal pellicle can impact oral health by influencing the initial colonisation of microbes on oral surfaces, maintaining mineral balance, protecting against acid damage, lubricating oral surfaces during daily activities, and preventing tooth fractures and mucosal irritation [49].

Saliva is a fluid produced by three major and minor salivary glands. It is a complex non-Newtonian fluid comprising 99% water and 1% organic and inorganic molecules. Proteins represent 0.3% of salivary composition; this protein content will vary depending on several factors such as age, disease, medication, medical treatments, food intake, and circadian rhythm.

Saliva contains proteins, enzymes, and electrolytes, including several ions. The contribution of the non-glandular composition is very significant in terms of saliva functions. Every salivary gland contributes to the secretion of unstimulated whole-mouth saliva (UWMS); it continuously flows reaching 500–600 mL daily, maintaining oral lubrication and protection. UWMS also contains eukaryotic cells (epithelial and leukocytic cells), microorganisms, and gingival–crevicular fluid (GCF) from the periodontal pocket [32,50].

UWMS interacts with oral tissue and continues flowing, developing a salivary film over oral mucosa. This leaves underneath a complex barrier, the mucosal pellicle, which plays a fundamental role in tissue protection, moistening, and lubrication [34,48,51].

In addition, saliva is a valuable non-invasive diagnostic fluid, offering significant advantages: it is easy to collect, non-invasive, and stress-free for the individual [27]. These features make it ideal for monitoring various conditions in humans, as it reflects biological processes occurring in the mouth during healthy and diseased states. Consequently, salivary molecules have been utilised as biomarkers for various local and systemic conditions [20,27,52].

Salivary proteins have been widely studied in health and disease using different techniques such as proteomics. Mass spectrometry (MS) and nuclear magnetic resonance (NMR) spectroscopy are the most common methods for studying salivary metabolites [23]. In contrast, fewer studies have focused on characterising and identifying salivary metabolites to use these molecules as a biomarker for diseases such as oral cancer, periodontitis, and Alzheimer’s disease [23].

The salivary flow rate and composition contribute to reducing the bacterial burden through mechanical clearance and immune surveillance. In addition, the growth of microbial communities is influenced by dietary nutrients and salivary proteins, particularly mucins. Between meals and during a low-sugar dietary supply, the breakdown of salivary proteins promotes microbial cooperation to obtain nutrients. This process releases oligosaccharides, which are digested, leading to lactate production, which can be converted into weaker acids, acetate and propionate, counteracting and neutralising the pH drop. This bacterial metabolic chain will form a food web that determines the spatial organisation of bacteria [21,53]. In addition, glycoproteins, peptides, and amino acids can be nutrients for bacteria on the tongue surface, breaking down into SCFAs, ammonia, and sulphur compounds, which have been associated with malodour-associated halitosis and periodontal disease [54,55].

In summary, the MP is integral to maintaining oral health by forming a protective layer that ensures tissue integrity, supports immune function, and regulates microbial activity. Its composition, influenced by saliva flow and protein content, plays a critical role in controlling microbial colonisation and maintaining a balanced oral environment. As research continues to unravel the complexities of salivary proteins and metabolites, we gain deeper insights into their potential as biomarkers for disease and their contributions to oral health maintenance.

## 3. Impact of Salivary Metabolites and Bacterial Dynamics on Oral Health

As previously mentioned, saliva and oral bacteria play a crucial role in maintaining the balance of the oral environment. Bacterial metabolites have been analysed in UWMS as an indicator of the patient’s oral and systemic health status due to its continuous daily flow. Therefore, metabolites can provide insights regarding microbial dysbiosis, indicating inflammation or diseases [12,17,34]. UWMS encompasses both endogenous and exogenous metabolites derived from various sources within the oral cavity, including gingival–crevicular fluid (GCF), microbial activity, and dietary intake. Additionally, systemic factors such as aging, systemic diseases, medication, and treatment for HNC can significantly influence the salivary metabolite profile [27,50,56].

The salivary microbiome and metabolome are highly dynamic due to the complexity of the oral cavity, which is constantly exposed to various influences. These include different foods (such as sugars), beverages, and interactions with other people and animals and are further impacted by factors like oral hygiene, stress, aging, obesity, physical activity, medications, sexually transmitted infections, pregnancy, and the surrounding environment, which encompasses socioeconomic, psychological, and environmental aspects [21,23,27,57].

Dietary changes can disrupt the balance and diversity of oral bacteria, leading to fluctuations in the metabolome that impact host–bacteria interactions, potentially causing inflammation and increasing disease risk. This can reveal altered biochemical pathways in the oral cavity [23], as studies show that glucose and sucrose exposure induce dynamic microbiome changes, promotes acid production, and decreases pH beyond the neutralising capacity of the saliva and oral bacteria [23,58]. This is particularly relevant during HNC treatment, as patients often experience significant weight loss and may require food supplements high in carbohydrates and sugars to help maintain their weight. This diet contains high levels of fermentable carbohydrates, such as sugar and starch, which serve as a food source for bacterial metabolism [59]. In addition, the intake and frequency of consumption are higher during this period, increasing the risk of oral diseases such as caries.

The mouth comprises a complex and diverse bacterial community with a critical role in oral health, in addition to host behaviour and defence determining the local environment, bacterial adherence, and virulence [37,60]. As we previously mentioned, salivary components determine microbiome balance and diversity, by promoting commensal bacteria’s function, which decreases opportunistic bacteria’s colonisation. Also, the composition of bacteria is shaped by the flow of saliva, which influences microbial adherence to surfaces. Saliva’s natural clearance and swallowing mechanisms continuously remove epithelial cells colonised by bacteria, leading to the ongoing recolonisation of mucosal surfaces [31,50,61,62]. Additionally, abrasion and oral hygiene practices help eliminate bacteria from non-shedding surfaces like enamel. The formation of the oral microbiome and biofilm is also influenced by dental restorations, prostheses, dentures, and implants [31].

Furthermore, reduced salivary flow can adversely affect bacterial composition by impairing clearance and allowing food residues to accumulate in the mouth. This altered environment compromises protective functions, disrupts microbiome diversity, and promotes the growth of pathogenic bacteria. Prolonged exposure to these conditions can result in dysbiosis, metabolic alterations, and an increased risk of pathology [17,20,43,49].

**Figure 3:**
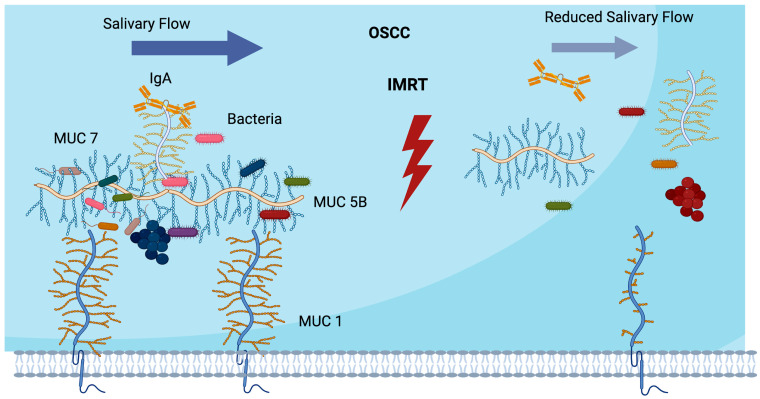
Radiotherapy effects. Mucosal pellicle breakdown during and after IMRT treatment would influence the mucosal barrier’s composition and function, impairing protection and lubrication, and reducing bacterial diversity and colonisation contributing to dysbiosis,. Similarly, a reduced salivary flow rate will impair microbial and food clearance increasing the risk of several oral diseases.

This is particularly relevant for HNC patients undergoing radiotherapy. Reports [20,52,63,64] suggest that IMRT can severely reduce the salivary flow rate and alter the protein composition in the salivary glands. These changes can disrupt bacterial composition, promote the colonisation of opportunistic and pathogenic bacteria on both hard and soft tissues, and compromise mucosal integrity (Figure 3). Consequently, this increases the risk of caries, periodontal disease, mucosal inflammation, and ulcers [20,37,60].

The resulting oral bacterial dysbiosis has also been linked to systemic diseases by disrupting homeostasis in distant sites throughout the body through harmful pathogens and inflammatory byproducts [65]; homeostasis results from the close interaction between a diverse and healthy microbiome and the immune system. However, a less varied and altered microbiota would lead to dysregulation, inflammation, dysbiosis, and disease onset [37,65,66]. Microbiome dysbiosis would impact several systemic diseases, such as immune-related diseases comprising rheumatoid arthritis, type 1 diabetes, multiple sclerosis and lupus erythematosus, inflammatory diseases, including Crohn’s disease and ulcerative colitis, inflammatory bowel disease, allergies, and asthma. The interplay between the host and microbes in the mouth highlights the crucial role of maintaining a balanced oral microbiome and healthy salivary flow and composition. Disruptions to this balance can lead to significant consequences that extend beyond the oral cavity, potentially impacting overall health [37,65,66]. Furthermore, dysbiosis can promote cancer development due the effect of periodontopathogens in the epithelium. *P. gingivalis* has a virulence factor known as gingipain, a cysteine protease. Gingipain promotes protein degradation in the tissue to allow bacterial infection and translocation. In addition, gingipain upregulates genes involved in the epithelial–mesenchymal transition via β-catenin [67,68]. Another virulence factor which promotes tumoural development is the cytolethal distending toxin secreted by *Aggregatibacter actinomycetemcomitan*. This toxin can produce DNA damage and even the cell cycle arrest mechanisms related to carcinogenesis [69].

Recently, researchers have understood that the microbiota could be improved and modified using probiotics (*Lactobacillus* and *Bifidobacterium*) to treat specific diseases, such as irritable bowel syndrome. In the same pattern, faecal transplants from healthy subjects have been used to rebuild the gut microbiota when conventional treatments are unsuccessful and in cases of *Clostridium difficile* infections. In addition, prebiotics (dietary fibres and oligosaccharides) are used to promote healthy gut bacteria, enabling diversity and homeostasis. On the other hand, postbiotics inhibit pathogenic bacteria and improve intestinal barrier function [37,65,66].

The interplay between the host and microbes in the mouth highlights the crucial role of maintaining a balanced oral microbiome and healthy salivary flow and composition. Disruptions to this balance can lead to significant consequences that extend beyond the oral cavity, potentially impacting overall health [37,65,66].

## 4. The Impact of Environmental and Host-Related Factors on the Oral Microbiome and Metabolites

Given the significant microbial differences observed between oral sites in HNC patients, it is crucial to consider the broader implications of these findings. The role of bacteria in carcinogenesis is intricately linked to their metabolic activity [70,71]. This shift in the bacterial community not only alters local inflammation but may also play a crucial role in cancer progression [70,71]. Therefore, the sample collection protocol of different oral sites must avoid contamination that would lead to confusion [57].

**Figure 4:**
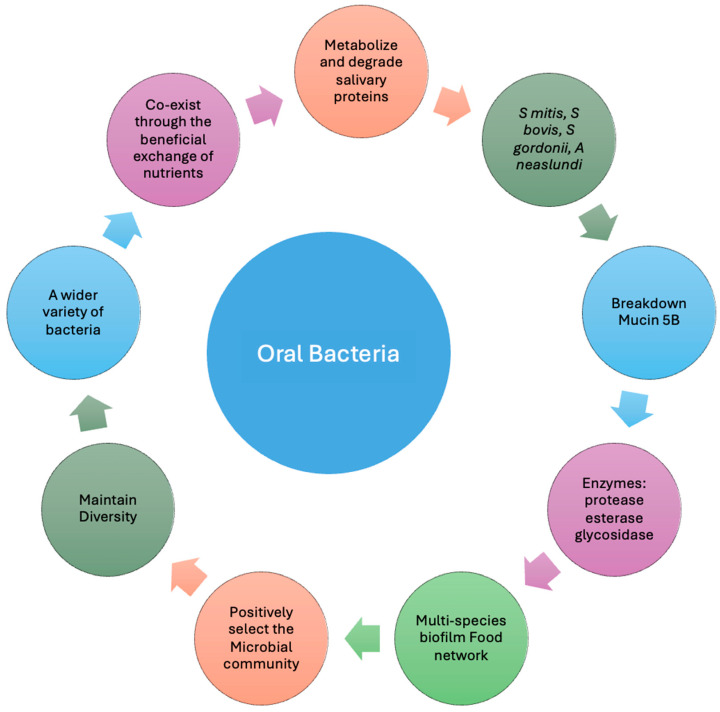
Homeostasis in oral bacteria maintained by glycoproteins as a source of nutrients. Mucins are glycoproteins present in saliva and mucosal pellicle; they are a source of nutrients for commensal bacteria and contribute to reducing adherence and colonisation, maintaining diversity, and allowing commensal microorganisms, excluding pathogenic bacteria, to impair dysbiosis associated with disease.

However, it is crucial to interpret the differences in microbial communities observed in HNC patients with caution, given the potential effects of smoking, alcohol consumption, and oral hygiene on the oral environment. These factors can significantly alter the oral microbiome, potentially confounding the results by altering bacterial surfaces and function and promoting the growth of certain bacteria to lead to an imbalance in microbial populations [37]. The effects of smoking on oral microbiota composition are associated with an increased abundance of anaerobic bacteria, salivary pH variation, and potential bacterial adhesion variations to oral surfaces. Additionally the toxic compounds of cigarettes can alter the host immune system response. All of these would alter microbiome diversity; however, contradictory results have been reported. In addition, smoking will contribute to the development of periodontitis and respiratory infections by increasing pathogenic bacteria and altering the microbiome composition. Similarly, alcohol consumption will reduce bacterial diversity, promoting epithelial permeability and inflammation, leading to dysbiosis and increasing the risk of oral diseases. It is well known that acetaldehyde resulting from alcohol metabolism in the oral cavity increases the risk of oral cancer by promoting inflammatory conditions [37,57,72], and mucins and other glycoproteins play a crucial role in the microbial ecosystem by acting as a source of nutrients (Figure 4). These substances, rich in sugars and carbohydrates, provide essential sustenance for bacteria. Moreover, salivary mucins will inhibit microorganism adherence and colonisation through the binding and aggregation of bacteria. In non-stimulated saliva, these nutrients are deficient and almost non-detectable, maintaining oral commensals [31,73,74]. In physiological conditions, bacteria offer many benefits to the host, which include obtaining nutrients from the proteolytic degradation of salivary proteins, producing some metabolites in saliva, defending against pathogens, and regulating host immunity [27].

As mentioned, IgA is crucial in preventing bacterial colonisation by blocking microbes from attaching to and invading mucosal epithelia [31,74]. It also agglutinates bacteria in solution by binding to bacteria such as *S. mitis*, *S. oralis*, and *S. mutans* [21,53] and to salivary mobile mucins that connect with MAM proteins anchored to the epithelial cells, specifically MUC 1, contributing to the formation of the mucosal pellicle layer [31,37].

The IgA concentration in saliva tends to increase in response to stress, infection, or inflammatory conditions. This increase helps to enhance mucosal immunity and protect against potential pathogens. Conversely, in a healthy oral environment, IgA levels in saliva are relatively stable and lower, playing more of a maintenance role, contributing to the equilibrium of the oral microbiome and preventing excessive bacterial colonisation [34,39,73].

Additionally, other environmental and intrinsic factors can impact the conformation of the oral microbiome and its organisational, defensive, and metabolic functions [31,73,74]. Oral bacteria can metabolise and degrade salivary proteins, especially glycoproteins; *S. mitis*, *S. bovis*, *S. gordonii*, and *Actinomyces neaslundii* have been shown to break down mucin 5b through specific enzymes such as protease, esterase activity, and glycosidase [75]. A multi-species biofilm supports a food network to positively select the microbial community, maintaining its diversity and allowing a wider variety of bacteria to co-exist through the beneficial exchange of nutrients. Some species will convert nitrate from the blood delivered to saliva into nitrite, associated with cardiovascular protection and a low caries risk because of increased salivary pH [76,77].

The catabolism and fermentation of carbohydrates produce SCFAs, including lactate, acetate, butyrate, propionate, and isobutyrate [78,79]. Oral bacteria generate SCFAs through the fermentation of amino acids. These end-metabolites play a critical role in shaping the oral microbiome by providing a competitive advantage to certain microorganisms over others [80]. Other dietary nutrients, such as bicarbonate and lactate, also serve as substrates for bacterial metabolism. Lactate has an anti-inflammatory effect and modulates the immune response in the oral cavity [81,82,83].

The disruption of epithelial barrier function can facilitate carcinogenesis and its progression. Additionally, SCFAs have been linked to promoting a suppressive immune response within the tumour microenvironment. This effect includes the downregulation of intercellular adhesion molecule-1 (ICAM-1) in oral squamous cell carcinoma, which helps the tumour evade effective antitumour immune responses [84]. ICAM-1 plays a crucial role in facilitating the adhesion of immune cells to the tumour site, and its reduced expression can hinder the recruitment and activation of antitumour immune cells, thereby allowing the tumour to evade immune surveillance and continue to progress [85]. Conversely, primary periodontal pathogens such as *Porphyromonas gingivalis* and *Fusobacterium nucleatum* have been extensively studied as critical contributors to periodontal disease and oral cancer [14]. The subgingival production of SCFAs, including butyric acid (BA), has been relatively underexplored. BA has been implicated in tissue destruction and the progression of periodontitis by affecting the junctional epithelium. It influences processes such as cell proliferation, migration, and attachment. The impact of BA on periodontal disease is concentration-dependent: high levels of BA are associated with tissue destruction, while lower concentrations may promote epithelial downgrowth, contributing to disease progression [82,86,87].

Arginine is one of the free amino acids in saliva obtained through the proteolysis of salivary and cellular proteins by bacterial and mammalian proteases. Arginine positively affects oral health, reducing caries risk by increasing the pH, favouring enamel remineralisation, and reducing mineral loss. In the oral cavity, arginolytic bacteria possess the arginine deiminase system (ADS), which converts arginine into ammonia, ATP, and CO_2_ [88]. Ammonia can increase the pH in the oral environment, contributing to developing a diverse and beneficial microbial community, reducing lactate-producing bacteria, maintaining homeostasis, reducing caries risk and mineral loss, and favouring tooth remineralisation [75,89].

On the other hand, the arginine concentration in the mouth may regulate biofilm structure, either promoting bacterial growth or disturbing biofilms, depending on this amino acid’s concentration. In addition, arginine’s role in metabolic pathways and microorganism-related processes and adaptation to changes in the oral environment has been explored. Gene expression affected by arginine affects bacterial adhesion and biofilm onset and development, varying the conformation, weight, and total number of microorganisms [90]. Furthermore, bacteria with robust arginine metabolism may interfere with pro-tumorigenic processes by depleting arginine, a key resource for tumour growth, and modulating immune responses [90]. Conversely, anaerobic bacteria that can mainly degrade ethanol from alcohol to acetaldehyde by fermenting alcohol, using the enzyme alcohol dehydrogenase, *Fusobacterium nucleatum,* could be involved in alcohol degradation, producing this carcinogenic molecule associated with a risk of oral cancer increased by alcohol consumption and poor oral hygiene [79,91].

Researchers observed a significant reduction in bacterial abundance in a study examining the oral microbiota of HNC patients undergoing IMRT at three different time points (pre-radiation, mid-radiation, and post-radiation). The predominant genera identified across all time points included *Streptococcus*, *Prevotella*, and *Veillonella*. Although there was a noticeable change in the alpha diversity indices between the pre- and post-radiation phases, no specific taxa were consistently identified across the time points. Additionally, alterations in the microbiome composition were associated with changes in the profile of secondary metabolites present in the saliva [92].

## 5. The Role of Microbial Dysbiosis and Metabolic Alterations in Cancer Progression

Variations in the microbiome composition are associated with oral and systemic diseases, including diabetes mellitus, cardiovascular diseases, and cancer. Microbial dysbiosis in different body sites can lead to systemic diseases through disruptions in the host–microbiota interaction by modulating the immune response [65,93].

Oral microbiome imbalance has been associated with systemic diseases such as irritable bowel syndrome and colorectal cancer (CRC) [94,95]. The *Porphyromonas* and *Fusobacterium* genera have been found in colorectal tumour samples [96], forming pivotal networks that act as bridge bacteria for colonisers. These bacteria are crucial in shaping microbial communities by altering barrier permeability and facilitating opportunistic microorganisms’ infiltration. This disruption leads to biofilm composition and organisation changes, promoting inflammatory processes that contribute to disease progression [97].

*Fusobacterium nucleatum* and *Porphyromonas gingivalis* have been shown to trigger a pro-inflammatory response in host tissues by elevating the levels of TNF-α, NF-κB, and IL-1β. Moreover, both bacterial species enhance their attachment to and invasion of host cells, increasing their potential to establish infections [70]. In addition to these effects, *Fusobacterium nucleatum* and *Porphyromonas gingivalis* promote the infiltration of immunosuppressive cells, such as regulatory T cells (Tregs) and myeloid-derived suppressor cells (MDSCs) [71]. This infiltration can significantly alter the local immune environment, impairing the function of immune effector cells, including cytotoxic T lymphocytes and natural killer (NK) cells [71]. Consequently, oral microbial persistence may also contribute to the progression of malignancies associated with chronic inflammation.

Moreover, in cancer onset and development, these bacteria may increase the number of cancer cells, several metabolites, and inflammatory factors that spread via the bloodstream [65,98]. Specifically in oral cancer, the literature revealed that the metabolic pathways of certain bacteria could influence metabolic processes regarding tumour progression and development. The bacteria found in oral cancer were *Streptococcus* and *Parvimona*. These bacteria were also associated with higher levels of IL-6 and TNF-α, both of which have a crucial role in cancer development, contributing to progression and inflammation [99].

Specific species, including *Aggregatibacter actinomycetemcomitans*, *Porphyromonas gingivalis*, *Prevotella intermedia*, *Campylobacter rectus*, *Peptostreptococcus micros*, *Treponema denticola*, and *Fusobacterium nucleatum*, have been implicated as key contributors to periodontal pathology [38]. Host inflammatory mediators are significantly altered, with notable increases in cytokines such as IL-1β, IL-6, and TNF-α observed in the saliva [38]. These cytokines are central players in the inflammatory response and have been linked to a broad spectrum of inflammatory diseases beyond periodontal conditions, including cardiovascular diseases, diabetes mellitus, and even systemic inflammatory responses.

Regarding oral cancer progression, this process is associated with increased levels of bacterial enzymes that break down fatty acids, such as carnitine O-palmitoyltransferase 1 (CPT1A). Additionally, increased levels of inflammatory and stress-related markers, including IL-6, oxidative-stress-responsive kinase 1 (OXSR1), and TNF-α, were observed in patients with oral cancer compared to healthy controls. These findings suggest a potential association between these enzymes’ presence and cytokines and oral cancer, implicating disrupted fatty acid metabolism indicated by reduced SCFA levels, oxidative stress, and altered immune responses. The study concludes that variations in the microbiota composition, modified fatty acid metabolic pathways, changes in inflammatory markers, and specific enzymes may serve as potential biomarkers for early oral cancer diagnosis [99]. Future research on the identification of bacterial signatures in oral cancer must integrate more detailed molecular insights with larger sample sizes and long-term monitoring to enhance the early detection and management of microbial factors associated with oral cancer.

**Figure 5:**
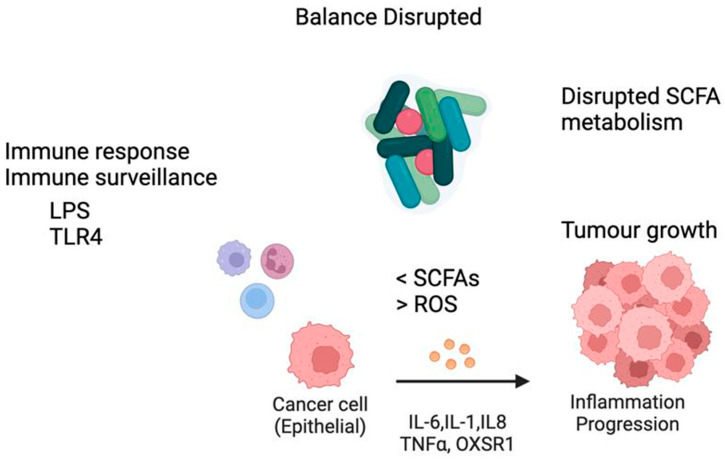
Microbiome dysbiosis and tumour pathogenesis. Disruptions in the oral microbiome lead to significant metabolic shifts within microbial communities. Pathogenic microorganisms linked to periodontitis produce virulence factors, such as gingipains and LPS. These factors compromise the epithelial barrier’s integrity, increasing its permeability and promoting tumor growth. The resulting changes create distinct microenvironments that drive inflammation by increased cytokines levels such as IL-6, IL-1. IL-8, and other, further contributing to tumor progression and disease pathogenesis.

Moreover, SCFAs, from bacterial products, modify DNA structure and affect the expression of specific genes by DNA methylation, regulating non-coding RNA and making certain genes more or less accessible for transcription. SCFAs can influence and affect immune cells and create a microenvironment that would encourage the growth of the tumour [57,100]. Cytokines and chemokines are key signalling molecules in the immune response, modulating the ability to target tumour cells. They also influence the production of reactive oxygen species (ROS) by bacteria, which can induce oxidative stress and cellular damage. This oxidative stress contributes to genetic mutations and facilitates cancer progression [71,93,100,101,102]. Other oral pathogens such as *P. gingivalis* can increase cytokine secretion, including IL-1, IL-6, IL-8, and TNF-α, which triggers inflammatory responses and can contribute to tumour progression [100]. Figure 5 summarises this process.

In the study conducted by Zhang et al. [93], researchers collected bilateral buccal mucosal tissues from the same patient diagnosed with oral squamous cell carcinoma (OSCC). They aimed to compare the microbiota present in 50 paired samples taken from tumour sites and non-tumour sites using 16S rDNA sequencing. The analysis revealed that 99.0% of the oral microbiota could be categorised into 13 phyla, indicating that almost all the identified oral bacteria fell within these major taxonomic groups. The predominant phyla included *Firmicutes*, *Proteobacteria*, *Bacteroidetes*, *Fusobacteria*, and *Actinobacteria*, which were present in both OSCC patients and healthy control groups. Despite the presence of oral cancer, there were significant overlaps in the microbial composition, indicating that certain bacteria are core components of the oral microbiome. This information may be important for understanding the role of the oral microbiota in health and disease, particularly in the context of OSCC.

The richness and diversity of bacterial populations were found to be significantly greater in tumour sites compared to control tissues. At the species level, there were marked increases in the abundances of *Fusobacterium nucleatum*, *Prevotella intermedia*, *Aggregatibacter segnis*, *Capnocytophaga leadbetteri*, *Peptostreptococcus stomatis*, and five other species, indicating a possible link between these bacteria and OSCC [93]. This dominance can manifest as an increased relative abundance of pathogenic species, which may decrease the levels of beneficial microorganisms, thereby altering the overall microbial diversity. Such shifts in microbial community structure can contribute to a dysbiotic state, promoting an inflammatory environment that may facilitate cancer progression.

Additionally, functional predictions indicated that genes related to bacterial chemotaxis, flagellar assembly, and LPS biosynthesis—processes associated with bacterial virulence—were significantly elevated in the OSCC group [93]. In this context, keystone microbes are those low-abundance taxa that, despite being few in number, may have critical roles in maintaining or disrupting the balance of the microbiome. The study [93] suggests that these low-abundance taxa may possess stronger virulence factors or pathogenic potential, meaning they could contribute significantly to disease processes, including cancer development.

The implication is that these keystone microbes could play a greater role in the progression or initiation of OSCC than their abundance alone might suggest. Their presence could influence the overall microbial community dynamics and immune responses or contribute to a pathogenic environment conducive to cancer.

Other studies found genera other than *Parvimonas*, associated with oral cancer, such as *Fusobacterium*, *Peptostreptococcus*, and *Neisseria,* which were elevated in oral cancer patients compared to healthy controls; additionally, increases in these bacteria were associated with the progression of oral cancer. Conversely, *Streptococcus* species were significantly reduced in cancer patients [93,103]. However, it is important to note that this cross-sectional study involved only 35 cancer patients. Many studies utilise observational or cross-sectional designs, which limit the ability to draw definitive conclusions about causality. Longitudinal studies and experimental designs, such as germ-free models or controlled microbiota manipulation, are needed to elucidate the mechanisms by which the microbiota may influence cancer development.

In the same pattern, the results of a different group, when comparing bacterial richness between cancer patients and healthy controls, reported that *Solobacteria, Peptostreptococcus*, and *Prevotella* were significantly more abundant in these patients [104]. Regarding salivary assessment, when comparing the stimulated whole-mouth saliva of HNC patients with healthy controls, the microbial profile was significantly different between the two groups. Pathogenic microorganisms such as *Streptococcus anginosus, Abiotrophia defective*, and *Fusobacterium nucleatum* were more abundant in HNC patients [105].

While numerous studies have established correlations between specific microbiota profiles and cancer development, it is crucial to approach these findings with a critical perspective. Correlation does not imply causation, and the relationship between microbiota and cancer is often multifaceted. One hundred twenty-four studies have been included in a systematic review [106] to evaluate the relation between the human microbiome and cancer. The gut microbiome was the microbiome most frequently studied, followed by the oral microbiome. Numerous bacteria inhabit the human oral cavity, which are linked not only to oral cancers but also to cancers of the lung, oesophagus, stomach, pancreas, and colorectum. Although the oral microbiome is thought to significantly contribute to carcinogenesis, its complexity—affected by various environmental and genetic factors—may lead to inconsistent findings in published research. The wide range of parameters utilised to characterise the microbial composition hindered the ability to standardise the various studies for meta-analysis.

Another study [15] investigated the compositional and metabolic profiles of the bacterial communities in different niches of oral cancer patients through the analysis of tumour surface and deeper tumour tissue samples, UWM saliva, and mucosal swabs. Specifically, tumour surfaces were found to be enriched with *Porphyromonas*, *Enterobacteria*, *Neisseria*, *Streptococcus*, and *Fusobacterium*, while deeper tumour tissues harboured *Prevotella*, *Treponema*, *Sphingomonas*, *Meiothermus*, and *Mycoplasma* [15]. The observation that microbial communities differ between the surface and core of tumour tissues indicates a high spatial variability in the microbial composition and the tumour microenvironment’s complexity.

Additionally, the tumour microenvironment influences the development of specific bacteria, with anaerobic species being particularly prevalent due to the hypoxic conditions present in tumours [70,71,93]. These variations contribute to tumour growth and invasion [15,57,93,107], promoting inflammation associated with cancer and potentially impairing the effectiveness of cancer treatments [15,57]. This environmental heterogeneity could support distinct microbial communities adapted to these specific conditions, such as the oxygen levels, nutrient supplies, and immune responses, by the presence of specific immune cells within the tumour. When the balance of the microbial community is disrupted, pathogenic bacteria can overgrow, leading to a chronic and uncontrolled inflammatory response.

Some inflammatory mediators comprise Toll-like Receptors (TLRs), NF-kB signalling, and ROS. This persistent inflammation can contribute to the malignant transformation of surrounding tissues, promoting carcinogenesis, tumour growth, and the epithelial–mesenchymal transition. It has been reported that a higher abundance of *Peptostreptococcus* can raise the TLR2 and TLR4 expression in colon cancer cells, increasing ROS and promoting cell production. On the other hand, *Porphyromonas gingivalis* and *Fusobacterium nucleatum* were associated with an altered immune response through interaction with TLRs on tumour cells promoting cancer cell proliferation by activating the NF-kB pathway [15,57,71]. Both oral-origin bacteria can keep the tumour cells away from immune system surveillance and clearance by promoting the infiltration of immunosuppressive cells and interfering with the function of immune killer cells [70,71].

Different microbiome metabolic pathways expressed in disease may play a role in tumour development, contributing to cancer progression [57,70,93,102,107]. Bacteria can secrete molecules that regulate the immune system, affecting the host’s capacity to control tumour growth. For instance, enhancing bacterial LPS biosynthesis can contribute to a pro-inflammatory microenvironment that triggers an immune response and chronic inflammation. The TLR4 can detect LPS from Gram-negative bacteria, activating the immune response to eradicate the pathogens as it follows. LPS would bind TLR4, activating a process of signalling activation, recruiting proteins MyD88 and TRIF activating downstream signalling pathways; this would lead to the nuclear translocation of NF-kB and generate pro-inflammatory cytokines. Therefore, a dysregulated TLR4 would promote chronic inflammation and would contribute to tumour cells evading the immune surveillance cells’ recognition, reducing the action of cytotoxic lymphocytes and NK cells [70,71,108].

Another critical aspect of inflammation involves bacterial chemotaxis and flagellar assembly, both of which are influenced by bacterial gene expression in tumour tissues. Chemotaxis is the process by which bacteria move towards chemical signals, while flagellar assembly provides them with the motility needed for movement. These mechanisms enhance the bacteria’s ability to invade and persist within the tumour microenvironment, thereby sustaining chronic inflammation [70,101]. *Fusobacterium nucleatum* is particularly notable for its increased virulence in oral cancer. It employs several strategies to evade the host immune response, including the upregulation of capsule biosynthesis, flagellum synthesis, and chemotaxis. These adaptations boost its motility and facilitate its invasion of various tumour sites.

The metabolic activity on the tumour surface is primarily associated with fatty acid biosynthesis, carbon metabolism, and amino acid metabolism. SCFAs have an essential role in immune regulation by preventing inflammatory conditions, also changing the oral microbiome’s structure and stimulating and inhibiting bacterial growth [109]. In this regard, high levels of *Prevotella* have been associated with cancer progression (colorectal, gastric, and oral cancer) by linking SCFA production to the hyperproliferation of cells in colorectal and oesophageal cancer. Similarly, *Campylobacter* has been associated with oesophageal adenocarcinoma progression and *Helicobacter pylori* with stomach cancer [15]. Furthermore, it has been determined high SCFA levels can blockade the antitumoural response, specifically the CTLA-4 blockade. Further studies need to be performed [110]. 

Moreover, the most abundant bacteria on tumour surfaces are *Porphyromonas*, *Enterobacteria*, *Neisseria*, *Streptococcus*, and *Fusobacterium*. In contrast, deeper tumour tissues show higher carbohydrate metabolism and organic polymer degradation activity. In these deep tissue samples, the most significant common genera are *Prevotella*, *Treponema*, *Sphingomonas, Meiothermus*, and *Mycoplasma*. The surface of tumours typically has a better oxygen and nutrient supply than the deeper regions, which can affect bacterial growth and metabolic activity. Deep tumour areas might experience hypoxia and nutrient deprivation, influencing the types of bacteria and metabolic pathways present. In summary, there are 24 different metabolic pathways between the surface of the tumour and deeper tumour tissue [15].

Moreover, bacteria can significantly influence host cell metabolic pathways by affecting essential cofactors required for enzymatic reactions and vitamin metabolism. By releasing specific metabolites, bacteria can alter the local environment, which impacts the availability and utilisation of these cofactors. This disruption can modify the metabolic activities of tumour cells, potentially accelerating tumour progression through effects on cell death and proliferation. Consequently, such shifts can lead to an increased number of cancer cells within the tumour microenvironment [70,71].

Regarding bacterial metabolic activity, cancer subjects exhibit the upregulation of iron transport mechanisms, tryptophanase activity, and superoxide dismutase. These changes indicate alterations in iron acquisition processes, shifts in amino acid metabolism associated with cancer, and increased protein degradation. This upregulation enhances nutrient availability and supports bacterial growth within the tumour microenvironment. Additionally, *F. nucleatum* boosts the production of haemolysins and adhesins and strengthens its iron transport system to support DNA synthesis and cellular respiration, thereby promoting microbial survival and proliferation within the tumour environment [101]. The latter is very important because it contributes to tumour onset and development through the dysregulation of the iron pathways in the epithelial cells caused by bacteria competing for iron availability to ensure their growth and subsistence. Additionally, this can increase oxidative stress in the tumour site [101].

In the case of amino acid metabolism, arginine and histidine degradation pathways are elevated in cancer patients compared with healthy subjects, and this is associated with microbial activity, specifically in the *Flavobacteriaceae* and *Peptostreptococcaceae* families. The microbial profiles of OSCC patients showed a higher relative abundance of bacteria such as *S. anginosus*, *A. defectiva*, *F. nucleatum*, and *Streptococcus anginosus* [102].

More studies are needed to establish these associations between the metabolic profile’s role in cancer development and progression. It has been suggested that dysbiosis would lead to malignant lesion onset or a microbial composition induced by tumour environment changes [71,107]. Therefore, changes in bacterial communities regarding type and quantities during OSCC onset and development could be used as a diagnostic signature and progression control for this disease, helping to detect high-risk patients. However, the cause–effect mechanism remains uncertain; OSCC research is not conclusive in this matter [71].

Longitudinal studies that integrate both microbial and metabolic profiles could provide valuable insights into the role of specific metabolic functions and bacterial communities in the development and progression of oral cancer. By tracking these changes over time, researchers may identify the key predictors of tumour initiation and progression, offering potential targets for early detection and intervention strategies.

## 6. The Effect of the Microbiome and Salivary Metabolites on HNC Therapy

The impact of the microbiome on cancer therapy, particularly chemotherapy, has been primarily studied in the gut. Research indicates that the microbiome composition can influence chemotherapy efficacy and progression through mechanisms such as immune regulation, microbial enzyme activity, and shifts in microbial ecology. These factors collectively affect the host’s response to treatment.

Saliva from oral cancer patients also exhibits a distinct bacterial community structure compared to that found in tumour tissues. Despite these differences, there are notable similarities in both taxonomic and metabolic profiles. For instance, UWM saliva from oral cancer patients showed increased levels of bacteria such as *Streptococcus*, *Lactobacillus*, and *Bacteroides* compared to tumour tissues, as well as enhanced acetoin biosynthesis [15]. These changes suggest that while the microbial composition of saliva differs from tumour tissue, certain bacterial taxa and metabolic activities are consistently elevated.

Bacterial metabolites can influence treatment efficacy and toxicity by modulating the immune response. Microbial enzymes and the immunoregulatory effects of bacteria can enhance the host’s metabolic capacity, thereby improving the efficacy of treatments, reducing their toxicity, and increasing the bioavailability of chemotherapy agents like irinotecan (CPT-11), particularly in cancers such as CRC entering a drug-tolerant persister state [111]. Bacteria can change different compounds’ pharmacokinetic parameters and pharmacodynamic characteristics in chemotherapy agents [57,112]. CPT-11 would change the gut bacteria’s microbial diversity, catalysis, and metabolism. Understanding this mechanism to maintain the microbiome’s diversity and ecology is essential to improve CPT-11 outcomes and the host immune response after chemotherapy to develop individualised therapies that would combine chemotherapy with a tailored dynamic atlas of gut bacteria [57,112].

After administering CPT-11 chemotherapy, a significant interplay occurs between the gut microbiota, the host’s immune environment, and the drug’s metabolism. This interaction is referred to as the “microbiota–host irinotecan axis”. It has been reported that decreased levels of SCFAs induced by CPT11 could aggravate this drug toxicity because of an altered epithelial and mucus layer and immune response [57,112]. Therefore, it is crucial to understand which bacteria would improve cancer treatment effectiveness and which would cause inflammatory reactions that would interfere with the therapy outcomes to develop tailored treatments based on the microbiome profile.

Mucositis and xerostomia are the most common oral complications of the non-surgical therapy of cancer [113]. In oral cancer treatment, radiotherapy and chemotherapy (cisplatin and carboplatin) cause mucosal toxicity as a side effect, resulting in radiation-induced oral mucositis (ROM) [57]. Oral mucositis is a severe affliction characterised by erythema, oedema, and ulcerations of the oral mucosa. However, the association between the oral microbiota and this side-effect toxicity is unclear. Some research links epithelial damage to changes in the microbiome, which may increase the risk of ROM. They propose that preserving bacterial balance through probiotics and dietary interventions could mitigate this effect by maintaining epithelial barrier integrity [57]. However, it is important to note that these findings are based on studies of the colon epithelium, not the oral mucosa.

Salivary gland hypofunction will produce an altered salivary flow rate and composition, which appears in the early days of cancer treatment [20]. More than 80% of HNC patients will suffer salivary gland hypofunction. Patients clinically report this side effect as xerostomia, a subjective dry mouth feeling [19,114]. However, salivary gland hypofunction and xerostomia treatment is primarily palliative, mainly by applying artificial lubrication and stimulating the salivary glands’ residual capacity when possible. In addition, radiation therapy will impair the saliva’s physical and rheological properties, making it stickier and viscous and turning it from transparent to yellow or brown [115,116]. Changes in the saliva’s physical properties have been connected to xerostomia, affecting mouthfeel perception by reducing lubrication and oral wetness [33,117].

Moreover, salivary gland hypofunction diminishes saliva’s protective functions, increasing the risk and accelerating the onset and development of oral disease. IMRT’s harmful effects on saliva result in a dry and fragile oral mucosa, oral mucosal discomfort, pain, and hampered speech. Regarding nutrition, altered saliva will alter taste perception, the formation and translocation of a food bolus, and mastication efficiency [19,114,118].

On the other hand, an altered flow rate and composition would impair oral homeostasis, increasing tooth demineralisation risk and reducing remineralisation due to the lack of calcium and phosphate [119,120]. In this scenario, HNC survivors are more prone to carious lesions, tooth fractures, or tooth loss, leading to tooth extractions, resulting in an increased risk of osteoradionecrosis of the jaw [121,122]. These oral health issues can significantly impact general health, daily functioning, social interactions, and psychological well-being. Consequently, patients may limit their daily activities and social engagements, which can severely deteriorate their quality of life [19,118,123,124,125].

In addition, it has been reported that epithelial barrier disruption would determine bacterial community variation [57] (Figure 3); this is relevant during cancer therapy when oral ulcerative mucosal toxicities occur due to radiation therapy, and ROM appears at a high rate (40–60% incidence) [20]. ROM presents an ulceration phase, which is the most important, associated with pain, weight loss, fatigue, apathy, and depression. Additionally, oral bacteria colonisation could aggravate mucositis, contributing to its length and severity by stimulating macrophages’ pro-inflammatory and additional cytokine secretion, affecting its resolution. Bacteria can colonise the lesions during the ulcerative phase, leading to secondary infection. The exact mechanism has not yet been elucidated; however, it is believed that bacteria cell wall products enter the submucosa, stimulating macrophages to secrete pro-inflammatory cytokines, developing inflammation [126,127]. Microbial homeostasis promotes healthy and functional epithelial barriers and anti-inflammatory factors that impede pathogen invasion. These metabolites produced by these bacteria would encourage epithelial barrier growth [57].

## 7. Conclusions and Future Directions

This article focuses on the role of the oral microbiome and salivary metabolites in maintaining oral health and their potential connections to systemic diseases and cancer progression. Understanding the interaction between salivary microbial metabolites and cancer therapy side effects can lead to more effective preventive and therapeutic strategies. These strategies would help manage the oral conditions that appear during and after the treatment.

Numerous studies have demonstrated that oral microbiota dysbiosis and disruptions in oral mucosal homeostasis can serve as modifiable risk factors for the development of OSCC. *Fusobacterium*, *Streptococcus*, *Peptostreptococcus*, *Porphyromonas gingivalis*, and *Prevotella* have been linked to oral carcinoma through mechanisms such as promoting cell proliferation, producing oncogenic compounds, and fostering an inflammatory microenvironment. While there is promising evidence connecting dysbiosis to an increased cancer risk, this area of research is still evolving.

The identification of specific bacterial species and their metabolites involved in oral cancer progression suggests that managing the oral microbiome could be a viable strategy for improving cancer treatment outcomes. In addition to focusing on metabolites, it is essential to consider the virulence factors of these microbial species, as they can significantly influence disease progression and the efficacy of treatments.

Improving our understanding of the specific metabolic pathways regarding oral microbiome balance and dysbiosis, particularly in the context of cancer, could lead to the development of tailored treatment methods (determined by specific patient condition) that enhance microbiome health to contribute to the efficacy and reduce the toxicity of cancer therapies.

Longitudinal studies are needed to establish metabolites and virulence factors as salivary biomarkers that could be used for the early diagnosis and monitoring of oral and systemic diseases. While the direct correlation between bacterial virulence factors in saliva and their use as biomarkers for early diagnosis or monitoring is still an area of ongoing research, the evidence suggests a promising link. Further studies are needed to validate these findings and establish reliable biomarkers for clinical use.

It is essential to note that oral bacterial communities can fluctuate over time due to various host factors, including sex, ethnicity, habitual exposure to OSCC risk factors (such as smoking, alcohol consumption, and betel nut chewing), and individual oral health conditions. Therefore, the careful control of variables related to inter-individual differences is crucial to accurately assess shifts in oral microbiome populations that are specifically attributable to early-stage tumourigenesis. As a result, while utilising these microbial and metabolic markers for early detection in clinical settings is not yet standard practice, their potential to enhance early diagnosis and intervention remains significant.

Monitoring and modulating the oral microbiome and metabolome could enhance treatment outcomes and reduce side effects such as oral mucositis, xerostomia, and radiotherapy caries to improve patients’ overall quality of life. Probiotics and dietary modifications that restore microbial balance and maintain epithelial integrity might mitigate treatment-related side effects and support overall oral health. Regular monitoring and intervention for oral complications in HNC survivors are crucial. Early detection and the management of issues like altered saliva flow, mucositis, and dental damage can prevent further complications and improve patient quality of life.
